# Sustained acute kidney injury as an independent risk factor for neurodevelopmental and growth outcomes in a single NICU center

**DOI:** 10.1186/s12887-024-04568-7

**Published:** 2024-04-02

**Authors:** Chen-Wei Yen, Ming-Chou Chiang, Shih-Ming Chu, Hsiao-Chin Wang, Li-Chun Wu, Po-Cheng Yen, Mei-Ching Yu

**Affiliations:** 1 Department of Pediatric Nephrology, Lin-Kou Chang Gung Memorial Hospital, Taoyuan, Taiwan; 2 Department of Pediatric General Medicine, Lin-Kou Chang Gung Memorial Hospital, Taoyuan, Taiwan; 3 Department of Neonatology, Lin-Kou Chang Gung Memorial Hospital, Taoyuan, Taiwan; 4 Department of Pediatrics, Shuang Ho Hospital, Taipei Medical University, New Taipei, Taiwan; 5 Department of Pediatrics, School of Medicine, College of Medicine, Taipei Medical University, Taipei, Taiwan; 6 Department of Neonatal Intensive Care Unit, Lin-Kou Chang Gung Memorial Hospital, Taoyuan, Taiwan; 7 Department of Pharmacy Administration, Lin-Kou Chang Gung Memorial Hospital, Taoyuan, Taiwan; 8 College of Medicine, Chang Gung University, Taoyuan, Taiwan; 9 Department of Pediatric Nephrology, Lin-Kou Chang Gung Memorial Hospital and College of Medicine, Chang Gung University, 5 Fusing Street, Gueishan, Taoyuan, 333 Taiwan

**Keywords:** Acute kidney injury, Neurodevelopment impairment, Early growth restriction, Neonatal intensive care unit

## Abstract

**Purpose:**

Acute kidney injury (AKI) is commonly seen in neonatal intensive care units (NICUs) and is potentially associated with adverse prognoses in later stages of life. Our study evaluated the impact of sustained AKI (SAKI) on both neurodevelopmental impairment (NDI) and early growth restriction (EGR) in neonates.

**Methods:**

This case-control study retrospectively analyzed the medical records of neonates diagnosed with SAKI in the NICU of a tertiary medical center during the period from January 2007 to December 2020. Cases without subsequent follow-up and those resulting in death were excluded. We analyzed demographic, biochemical, and clinical outcome data.

**Results:**

Of the 93 neonates with SAKI, 51 cases (54.8%) were included in this study, while 42 cases (45.2%) were excluded due to a lack of follow-up or death. An age-matched control group comprised 103 neonates, who had never experienced AKI or SAKI, were selected at random. In total, 59 (38.3%) cases were identified as NDI and 43 (27.9%) as EGR. Multivariate analysis revealed that patients with SAKI had significantly higher risks of developing NDI (odds ratio, [OR] = 4.013, *p* = 0.001) and EGR (OR = 4.894, *p* < 0.001). The AKI interval had an area under the receiver operating characteristic curve of 0.754 for NDI at 9.5 days and 0.772 for EGR at 12.5 days.

**Conclusions:**

SAKI is an independent risk factor for both NDI and EGR in neonates. Consequently, regular monitoring, neurological development assessments, and appropriate nutritional advice are crucial to these infants who have experienced renal injury.

## What is known

- Acute kidney injury (AKI) is not rare in neonates hospitalized in intensive care units.
- Neonatal AKI increases mortality and prolongs hospital length of stay. Neonatal AKI is also potentially associated with adverse prognoses later in life in both full- and preterm neonates, such as increases the risks of elevated blood pressure and chronic kidney disease.

## What is new

- The occurrence of sustained AKI in full- and preterm neonates admitted to intensive care units can potentially result in adverse growth and neurological outcomes later in life.
- An extended duration of sustained AKI, particularly exceeding 9.5 days after birth, increases the likelihood of future neurological, growth, and developmental sequelae. Therefore, comprehensive monitoring and optimal interventions should be considered integral components of the management strategy for this vulnerable pediatric population as they grow and develop.

## Introduction

Acute kidney injury (AKI) is a medical condition characterized by a sudden decline in kidney function, leading to the accumulation of waste products such as urea, imbalances in electrolyte levels, and disruptions in fluid balance and acid-base homeostasis [1, 2]. This condition is of a significant concern in neonates, newborns within the first 28 days of life, especially those who are critically ill [1, 3, 4].

 Previous studies have shown that the incidence of AKI among neonates in Neonatal Intensive Care Units (NICUs) is quite variable but alarming, with rates ranging from 2.4 to 70% [2–7]. Several factors contribute to this wide range, including the population studied, diagnostic criteria used, and the severity of other underlying health conditions. Neonatal AKI is not only an immediate concern but also has long-term implications [1, 4–8]. Numerous studies suggest that neonates, whether full-term or preterm, who experience AKI are at greater risk of facing adverse outcomes later in life. These may include prolonged hospital length of stay (LOS), increased mortality rates, elevated blood pressure, and a higher likelihood of developing chronic kidney disease (CKD) [1–9].

In addition to the more commonly discussed outcomes, research has begun to shed light on other long-term consequences of AKI in neonates. Specifically, risk factors for neurodevelopmental impairment (NDI) and early growth restriction (EGR) have been identified [10]. These issues are particularly important because early neurodevelopment and growth trajectories can have a profound impact on a child’s quality of life, cognitive development, and even future educational attainment [11, 12].

Therefore, in this study, we aim to investigate the influence of sustained AKI (SAKI) on NDI and EGR in neonates hospitalized in NICUs. The results could offer actionable insights for clinicians working in these settings. By gaining a better understanding of how SAKI interacts with neurodevelopmental and growth outcomes, medical teams may implement more effective treatment options and long-term care plans.

## Materials and methods

### Study population

This case-control study retrospectively examined the medical records of neonates admitted to the NICU immediately after birth at Chang Gung Memorial Hospital, the largest tertiary neonatal care center in Taiwan, during the period from January 1, 2007 to December 31, 2020. This study was approved by the Institutional Review Board of Chang Gung Memorial Hospital in Taiwan (Institutional Review Board no. 201900820A3C101). All methods were performed in accordance with the relevant guidelines and regulations. Data were collected, reviewed, de-identified, and anonymized before analysis, and the ethics committee waived the requirement for informed consent because of the anonymized nature of the data and scientific purpose of the study.

All medical records of neonates admitted to the NICU post-birth were reviewed, with a detailed review conducted for those requiring pediatric nephrology consultation. Our study included patients diagnosed with SAKI, excluding those who lacked follow-up or had deceased. The control group participants were required to meet the following criteria: no history of AKI or non-SAKI AKI during NICU hospitalization post-birth and a minimum of 2 years of follow-up post-discharge. It was also imperative that the proportions of preterm infants, gestational ages, and gender ratios were closely matched between the SAKI and control groups. Mr. P-C Yen, not a physician and uninformed of the study’s purpose beforehand, randomly selected eligible neonates hospitalized in the NICU from January 1, 2015, to December 31, 2018, as control subjects, in accordance with these criteria.”

### Data collection

The medical records of all eligible subjects provided the following data: gestational age, gender, birth head circumference (HC), birth body weight (BW), birth body height (BH), APGAR score, intrauterine growth restriction (IUGR) status, incidence of neonates requiring renal replacement therapy (RRT) during AKI, duration of AKI, hospital length of stay (LOS), and total daily caloric intake (kcal/kg/day). Laboratory data documented included serum hemoglobin levels and serum HCO3- concentrations in preterm neonates at postmenstrual age (PMA) of 40 weeks and in term neonates prior to discharge. Additionally, the definitions of the following terms were provided: neonatal AKI criteria, SAKI and its recovery, non-congenital urological anomalies, perinatal asphyxia, bronchopulmonary dysplasia and its severity, brain insult and severe intraventricular hemorrhage, and neurodevelopmental impairment and early growth restriction.

### Neonatal AKI criteria

Changes in serum creatinine (SCr) or urine output (UO) were used to establish a diagnosis of neonatal AKI based on consensus and the modified neonatal Kidney Disease Improving Global Outcomes (KDIGO) AKI definitions [2, 3, 13, 14]. In our institute, SCr is measured using the colorimetric method Jaffe reaction. The UO is recorded in every neonate in our NICU.

### Definition of SAKI and recovery from AKI

SAKI is defined as AKI persisting > 7 days. Recovery from AKI is defined as one of the following: SCr level dropped to baseline at infancy or the initial level [15]; UO amount ≥ 0.5 mL/kg/h over 6 consecutive hours [6, 16]; and RRT discontinued or no longer needed.

### Non-congenital urological anomalies

In our study, subjects might have been diagnosed with prenatal or postnatal abnormalities affecting various organs. These include maldevelopment of the skull bone, primary hypothyroidism, auditory dysfunction, microcolon, buttock teratoma, gastroschisis, left diaphragm eventration, cleft palate, and imperforate anus. Anomalies that arose from specific events, such as postoperative short bowel syndrome due to necrotizing enterocolitis, bacterial meningitis, and neonatal stroke, were categorized as acquired anomalies. None of the subjects in our study had congenital malformations of the urinary system.

### Definition of perinatal asphyxia

According to the report by the American College of Obstetricians and Gynecologists, Perinatal asphyxia is defined as an acute hypoxic-ischemic event occurring in term and late preterm infants (gestational age ≥ 35 weeks) and includes: (1) an APGAR score of less than 5 at 5 and 10 min; (2) a fetal umbilical artery pH of less than 7.0, a base deficit greater than or equal to 12 mmol/L, or both; (3) brain injury evident on brain magnetic resonance imaging consistent with acute hypoxia-ischemia; or (4) the presence of multi-organ failure consistent with hypoxic-ischemic encephalopathy [17].

### Definition of bronchopulmonary dysplasia (BPD) and its severity

Clinically, BPD is defined as a persistent requirement for supplemental oxygen and/or respiratory support at either 28 days postnatal age or 36 weeks PMA in a preterm neonate (gestational age < 32 weeks) with radiographic evidence of parenchymal lung disease [18]. According to the 2019 Jensen definition, BPD severity at 36 weeks PMA is determined by the type of respiratory support needed, irrespective of FiO2 levels. The categories are: (1) Mild BPD (Grade I), where patients require low flow nasal cannula (< 2 L/min); (2) Moderate BPD (Grade II), where patients need continuous positive airway pressure, nasal intermittent positive pressure ventilation, or nasal cannula flow of ≥ 2 L/min; (3) Severe BPD (Grade III), where patients require invasive mechanical ventilation [18].

### Definition of brain insult and severe intraventricular hemorrhage (IVH)

Brain insults during the perinatal period have been identified as potential contributors to NDI [19–22]. In this context, a brain insult is defined as perinatal asphyxia, which may occur with or without resuscitation and with or without hypoxic-ischemic encephalopathy. Regarding the severity of IVH, using the Papile classification, grades III and IV are classified as severe [23, 24].

### Definition of neurodevelopmental impairment (NDI) and early growth restriction (EGR)

Neurodevelopmental assessment in preterm neonates and infants with abnormalities during hospitalization after birth typically encompasses physical, cognitive, behavioral, emotional, and social development [25, 26]. NDI is defined as the failure to achieve developmental milestones, which may or may not be accompanied by a smaller HC for age. The age for neurodevelopmental assessment is based on chronological age for term births and corrected age (CA) for preterm births [27].

NDI is confirmed by a pediatric psychiatrist or pediatric rehabilitation physician using the Bayley Scales of Infant and Toddler Development, Third Edition (Bayley-III) during outpatient follow-up [28]. The Bayley-III assesses three composite indices: cognitive, language, and motor, with age-standardized scores calculated using test norms (mean of 100 and standard deviation [SD] of 15). A score below 85 indicates developmental delay [28]. In Taiwan, infants undergo neonatal health screenings five times: at birth to 2 months, and at 2–4, 5–9, 10–18, and 19–24 months of age [29]. EGR is defined as having at least one of BH, BW, or HC below the 3rd percentile on national growth charts at a chronological age of 2 years for term births and a CA of 2 years for preterm births [29].

### Clinical outcomes

To evaluate long-term outcomes, clinical events were documented, including the Bayley-III scores in 18–24 months old infants and numbers of neonates diagnosed with cerebral palsy and needing long-term RRT. In addition, BH, BW, and HC were recorded at 6 months, 1 year, and 2 years. To assess whether SAKI is an independent risk factor for NDI, EGR, or both, we adjusted for the effects of multiple confounders on the outcomes of interest, including the APGAR score, brain insult in the perinatal period, non-congenital urological anomalies, SAKI, and need for RRT during AKI.

The area under the receiver operating characteristic curve (AUROC) was used to identify optimal cutoffs of the relevant parameters for predicting relationships between the duration of AKI and onset of NDI and EGR in neonates admitted to the NICU.

### Statistical analysis

Descriptive statistics are presented for clinical characteristics. Continuous variables were analyzed using parametric and nonparametric tests as appropriate. Results are reported as mean ± SD for continuous parametric data and median with interquartile range (IQR) for nonparametric data. Student’s t-test and the χ^2^ test with Fisher’s exact test were used to examine the significance of differences in continuous and categorical variables between groups, respectively. All covariates were examined in univariate analyses. Multivariate logistic regression analyses were performed to identify probable risk factors for NDI, EGR, and both. Variables were retained in the final model if the *p*-value was < 0.05. All statistical analyses were performed using SPSS for Window software (ver. 26.0; IBM Corp., Armonk, NY, USA). For all analyses, a two-tailed *p*-value < 0.05 was considered statistically significant.

## Results

### Clinical characteristics and serum laboratory parameters between neonates in the SAKI and control groups

**Fig. 1:**
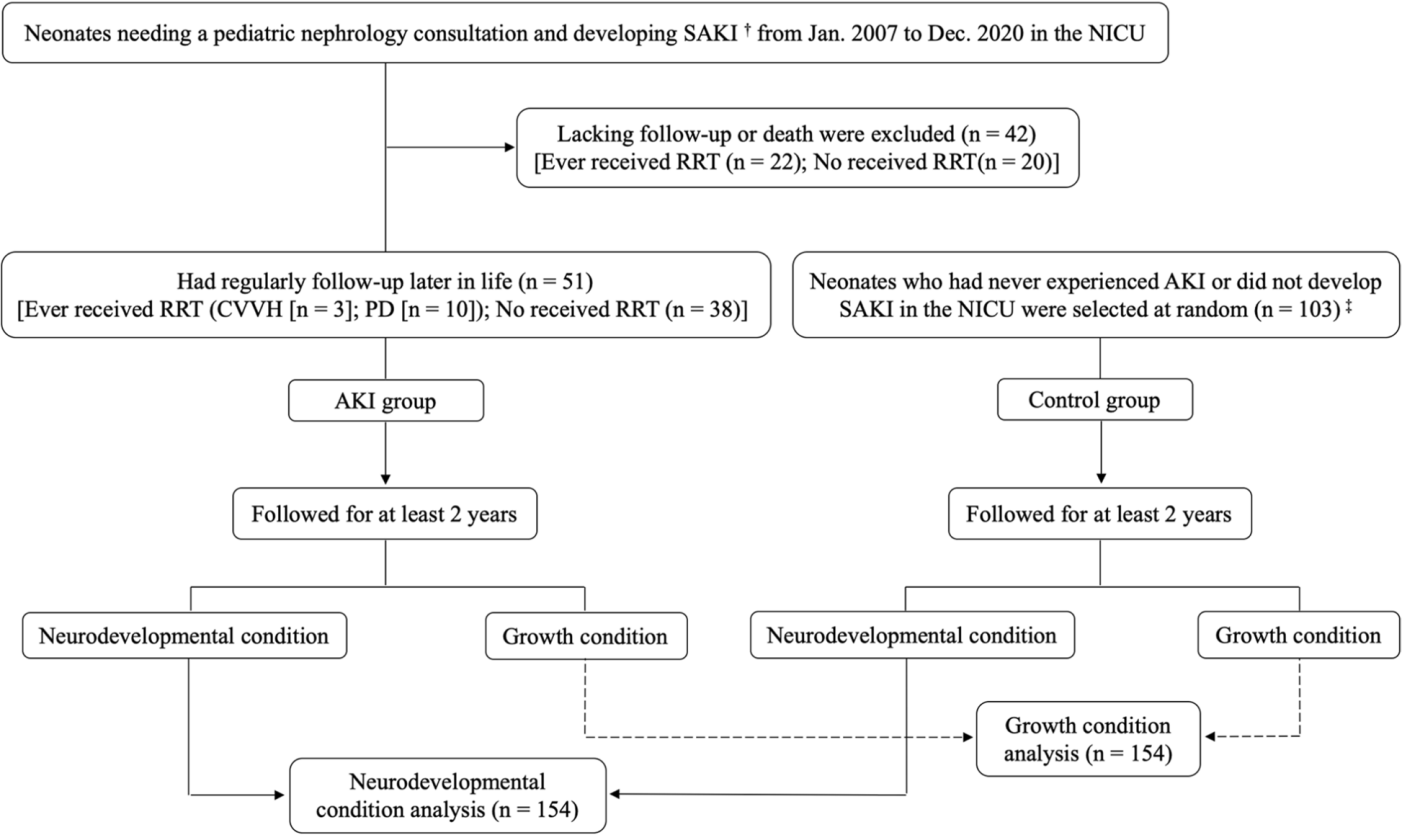
Flow diagram of participant selection. SAKI: Sustained acute kidney injury; NICU: Neonatal intensive care unit; RRT: Renal replacement therapy; CVVH: Continuous veno-venous hemofiltration; PD: Peritoneal dialysis; AKI: Acute kidney injury. †: SAKI: Acute kidney injury persisted longer than 7 days. ‡: Neonates who had never experienced AKI or did not develop sustained AKI in the NICU were randomized to the control group from 1 January 2015 to 31 December 2018. The proportion of preterm infants, gestational age and gender ratio were closely similar to those in the SAKI group

The study identified 334 neonates hospitalized in the NICU who underwent a pediatric nephrology consultation. Of these, 93 (27.8%) who met the neonatal modified KDIGO criteria for AKI developed SAKI. Over the 13-year study period, a total of 13,073 neonates were admitted to the NICU post-birth, with an incidence rate of SAKI at 0.71%. After excluding 42 (45.2%) neonates due to a lack of follow-up or death, the remaining 51 (54.8%) were included in the SAKI group. As controls, 103 neonates were selected at random, 77 of whom had never experienced AKI and 26 who had AKI but not SAKI. The neurodevelopment and growth of the patients in the SAKI group (*n* = 51) and the control group (*n* = 103) were analyzed (Fig. 1).

The mean gestational age was 32.6 ± 5.2 weeks, the mean birth BH was 42.0 ± 6.9 cm, the mean birth BW was 1.90 ± 0.93 kg, the mean birth HC was 29.4 ± 4.2 cm, and the mean APGAR score at 1 and 5 min after birth was 6.2 ± 2.4 and 8.1 ± 1.7, respectively. There were 109 (70.8%) preterm neonates, including 86 (55.8%) males and 42 (27.3%) with IUGR. Thirteen (16.9%) neonates needed RRT during AKI; the mean duration of AKI persisted was 12.2 ± 18.9 days, and the median (IQR) hospital LOS was 38.5 (19.0–84.0) days. In preterm neonates, the serum hemoglobin was 11.5 ± 1.3 g/dL, serum HCO_3_^−^ concentration was 23.9 ± 3.1 mmol/L, and total daily calories at 40 weeks of PMA was 115.3 ± 14.3 kcal/kg/day. In term neonates, the respective values were 12.2 ± 2.1 g/dL, 24.1 ± 2.6 mmol/L, and 10.4.9 ± 5.6 kcal/kg/day (Table 1).

**Table 1:** Comparison of clinical characteristics and serum laboratory parameters between neonates in the SAKI and control groups

	Total (*n* = 154, 100%)	SAKI group (*n* = 51, 33.1%)	Control group (*n* = 103, 66.9%)	* P* value
Gestational age (weeks)	32.6 ± 5.2	32.6 ± 5.2	32.6 ± 5.2	0.986
Preterm birth, n (%)	109 (70.8%)	36 (70.6%)	73 (70.9%)	0.971
Late preterm birth, n (%)	30 (27.5%)	10 (27.8%)	20 (27.4%)	0.978
Male gender, n (%)	86 (55.8%)	32 (62.7%)	54 (52.4%)	0.228
Birth body height (cm)	42.0 ± 6.9	41.6 ± 7.0	42.1 ± 6.9	0.684
Birth body weight (kg)	1.90 ± 0.93	1.88 ± 0.89	1.91 ± 0.96	0.848
Birth head circumference (cm)	29.4 ± 4.2	29.6 ± 4.1	29.3 ± 4.3	0.745
1-min APGAR score	6.2 ± 2.4	5.2 ± 2.7	6.7 ± 2.1	< 0.001
5-min APGAR score	8.1 ± 1.7	7.4 ± 2.0	8.5 ± 1.4	< 0.001
Intrauterine growth restriction, n (%)	42 (27.3%)	13 (25.5%)	29 (28.2%)	0.729
Brain insult in the perinatal period ^a^, n (%)	26 (16.9%)	10 (19.6%)	16 (16.8%)	0.014^*^
IVH	22 (14.3%)	7 (13.7%)	15 (14.6%)	0.890
Resolved IVH, n (%)	18 (11.7%)	4 (7.8%)	14 (13.6%)	0.299
Severe IVH ^b^, n (%)	4 (2.6%)	3 (5.9%)	1 (1.0%)	0.072
Epilepsy needing AED treatment ^c^, n (%)	12 (7.8%)	5 (9.8%)	7 (6.8%)	0.515
Non-congenital urological anomalies ^d^, n (%)	15 (9.7%)	10 (19.6%)	5 (4.9%)	0.003^*^
Needing RRT during AKI, n (%)	13 (16.9%)	13 (25.5%)	0 (0%)	< 0.001
Duration of AKI (days)	12.2 ± 18.9	16.6 ± 22.0	3.6 ± 1.2	0.004^*^
PMA at 40 weeks in preterm neonates				
Serum hemoglobin (g/dL)	11.5 ± 1.3	11.3 ± 1.1	11.6 ± 1.4	0.223
Serum HCO_3_^−^ (mmol/L)	23.9 ± 3.1	22.7 ± 3.4	24.5 ± 2.8	0.006^*^
Total calories (kcal/kg/day)	115.3 ± 14.3	115.5 ± 18.0	115.2 ± 12.1	0.911
Before discharge from hospital in term neonates				
Serum hemoglobin (g/dL)	12.2 ± 2.1	11.4 ± 1.6	12.6 ± 2.3	0.079
Serum HCO_3_^−^ (mmol/L)	24.1 ± 2.6	24.0 ± 3.7	24.1 ± 1.9	0.927
Total calories (kcal/kg/day)	104.9 ± 5.6	110.3 ± 15.3	102.1 ± 15.2	0.095
Number of neonates had BPD, n (%)	49 (31.8%)	12 (23.5%)	37 (35.9%)	0.122
Moderate (grade II) BPD, n (%)	12 (7.8%)	4 (7.8%)	8 (7.8%)	0.987
Severe (grade III) BPD, n (%)	1 (0.6%)	1 (1.9%)	0 (0%)	0.156
Hospital length of stay (days)	38.5 (19.0–84.0)	51.0 (23.5–132.0)	32.0 (14.0–67.5)	< 0.001

Data are expressed as the mean ± standard deviation or as the median (interquartile range, IQR). Statistical significance is established at a *P- value of < 0.05

^a^Perinatal asphyxia with or without resuscitation/with or without hypoxic ischemic encephalopathy

^b^Severe IVH [23, 24]: IVH grade III and IV

^c^Had epileptiform discharge on electroencephalography needing long-term antiepileptic drug treatment

^d^Maldevelopment of skull bone, primary hypothyroidism, auditory dysfunction, microcolon, buttock teratoma, gastroschisis, left diaphragm eventration, cleft palate, imperforated anus, post operation related short bowel syndrome due to necrotizing enterocolitis, bacterial meningitis and neonatal stroke

Compared to the control group, the SAKI group had lower 1- and 5-min APGAR scores (both *p* < 0.001), a higher percentage of brain insults in the perinatal period (*p* = 0.014), more non-congenital urological anomalies (*p* = 0.003), more patients needing RRT during AKI (*p* < 0.001), longer duration of AKI (*p* = 0.004), and longer hospital LOS (*p* < 0.001). There were no significant differences in gestational age, number of preterm neonates, gender, birth BH, BW, HC, number of neonates with IUGR, IVH, or severe IVH, percentage of epilepsy cases needing antiepileptic drug treatment, or proportion of bronchopulmonary dysplasia between the two groups (Table 1).

### Long-term outcomes of neonates in the SAKI and control groups

In the neurodevelopmental evaluation, more neonates in the SAKI group had NDI (*p* < 0.001) and significantly lower Bayley-III cognitive (*p* = 0.042), language (*p* = 0.004), and motor (*p* = 0.019) scores when while they were 18–24 months old. There was no group difference in the proportion of patients diagnosed with cerebral palsy (Table 2).

**Table 2:** Comparisons of long-term neurodevelopmental and growth outcomes in neonates with SAKI episodes versus controls

	Total (*n* = 154, 100%)	SAKI group (*n* = 51, 33.1%)	Control group (*n* = 103, 66.9%)	*P* value
Neurodevelopmental impairment, n (%)	59 (38.3%)	33 (64.7%)	26 (25.2%)	< 0.001
Bayley Scales of Infant and Toddler Development ^a^				
Cognitive score (n/total)	91.0 ± 14.8 (85/154)	86.7 ± 13.2 (29/59)	93.4 ± 15.1 (56/103)	0.042^*^
Language score (n/total)	87.1 ± 14.0 (85/154)	80.7 ± 12.4 (29/59)	90.1 ± 13.9 (56/103)	0.004^*^
Motor score (n/total)	88.8 ± 17.0 (85/154)	82.8 ± 16.0 (29/59)	91.9 ± 16.8 (56/103)	0.019^*^
Cerebral palsy, n (%)	4 (2.6%)	3 (5.9%)	1 (1.0%)	0.072
Early growth restriction, n (%)	43 (27.9%)	27 (52.9%)	16 (15.5%)	< 0.001
Body height (cm)				
At the CA of 6 months	64.2 ± 3.7	62.3 ± 4.5	65.0 ± 2.9	< 0.001
At the CA of 1 year	72.8 ± 3.6	70.9 ± 4.5	73.5 ± 2.9	< 0.001
At the CA of 2 years	84.0 ± 4.6	81.6 ± 5.9	85.0 ± 3.7	0.001^*^
Body weight (kg)				
At the CA of 6 months	7.07 ± 1.22	6.45 ± 1.42	7.34 ± 1.02	< 0.001
At the CA of 1 year	8.77 ± 1.53	7.85 ± 1.76	9.17 ± 1.23	< 0.001
At the CA of 2 years	11.22 ± 1.72	10.12 ± 1.56	11.63 ± 1.60	< 0.001
Head circumference (cm)				
At the CA of 6 months	41.6 ± 1.9	40.5 ± 2.2	42.0 ± 1.6	< 0.001
At the CA of 1 year	44.3 ± 4.1	43.5 ± 2.2	44.7 ± 4.6	0.148
At the CA of 2 years	46.9 ± 2.0	45.8 ± 2.2	47.3 ± 1.8	0.003^*^
Simultaneous NDI and EGR, n (%)	15 (9.7%)	10 (19.6%)	5 (4.9%)	0.003^*^
Needing long-term RRT, n (%)	4 (5.2%)	4 (7.8%)	0 (0%)	0.004^*^

Data are expressed as the mean ± standard deviation and a *P-value of < 0.05 is considered statistically significant

^a^Third Edition (Bayley-III) in 18–24 months old

Early growth was evaluated using BH, BW, and HC at ages or CA of 6 months, 1 year, and 2 years. In the SAKI group, BH was shorter, BW was lower, and HC was smaller. More neonates in the SAKI group had simultaneous NDI and EGR and needed long-term RRT over the 2 consecutive years of observation (Table 2).

In the univariate analyses (Table 3), NDI was associated with lower 1- and 5-min APGAR scores (odds ratio [OR] = 0.192, *p* < 0.001; OR = 0.212, *p* < 0.001, respectively), SAKI (OR = 5.429, *p* < 0.001), and needing RRT during AKI (OR = 6.259, *p* = 0.007). EGR was correlated with SAKI (OR = 6.117, *p* < 0.001) and needing RRT during AKI (OR = 7.081, *p* = 0.002). Simultaneous NDI and EGR was related to SAKI (OR = 6.021, *p* < 0.001) and needing RRT during AKI (OR = 6.667, *p* = 0.002).

**Table 3:** Logistic regression analysis of risk factors for NDI, EGR, and concurrent NDI and EGR in neonates with various medical conditions in the NICU

	Univariate			Multivariate		
	OR	95% CI	*P* value	OR	95% CI	*P* value
**Neurodevelopmental impairment (NDI)**						
1-min APGAR score ^a^	0.192	0.094–0.391	< 0.001	0.440	0.146–1.324	0.144
5-min APGAR score ^b^	0.212	0.105–0.430	< 0.001	0.408	0.134–1.244	0.115
Brain insult in the perinatal period	1.008	0.424–2.397	0.986			
Non-congenital urological anomalies	2.670	0.898–7.938	0.077			
SAKI	5.429	2.626–11.225	< 0.001	4.013	1.702–9.459	0.001^*^
Needing RRT during AKI	6.259	1.645–23.805	0.007^*^	2.217	0.467–10.514	0.316
**Early growth restriction (EGR)**						
1-min APGAR score ^a^	0.965	0.470–1.981	0.922			
5-min APGAR score ^b^	1.038	0.506–2.131	0.919			
Brain insult in the perinatal period	0.415	0.134–1.284	0.127			
Non-congenital urological anomalies	1.329	0.427–4.141	0.624			
SAKI	6.117	2.844–13.157	< 0.001	4.894	2.133–11.229	< 0.001
Needing RRT during AKI	7.081	2.050–24.454	0.002^*^	2.500	0.655–9.537	0.180
**Simultaneous NDI and EGR**						
1-min APGAR score ^a^	0.678	0.298–1.546	0.356			
5-min APGAR score ^b^	0.724	0.318–1.650	0.443			
Brain insult in the perinatal period ^#^	0.327	0.073–1.473	0.145			
Non-congenital urological anomalies	1.742	0.511–5.937	0.375			
SAKI	6.021	2.550–15.083	< 0.001	4.821	1.832–12.681	0.001^*^
Needing RRT during AKI	6.667	2.039–21.802	0.002^*^	2.528	0.698–9.157	0.158

*OR* Odds ratio, *CI* Confidence interval

^*^A *P*-value of < 0.05 is considered statistically significant

^a^Median number of 1-min APGAR score (7 point) used for logistic regression analysis

^b^Median number of 5-min APGAR score (9 point) used for logistic regression analysis

^#^Perinatal asphyxia with or without resuscitation/with or without hypoxic ischemic encephalopathy

In the multivariate logistic regression, SAKI was the only factor remaining in the final model and was independently related to NDI (OR = 4.013, *p* = 0.001), EGR (OR = 4.894, *p* < 0.001), and simultaneous NDI and EGR (OR = 4.821, *p* = 0.001) (Table 3).

### Predictive power of the relationships between the duration of AKI and the onset of NDI and EGR in neonates

**Fig. 2:**
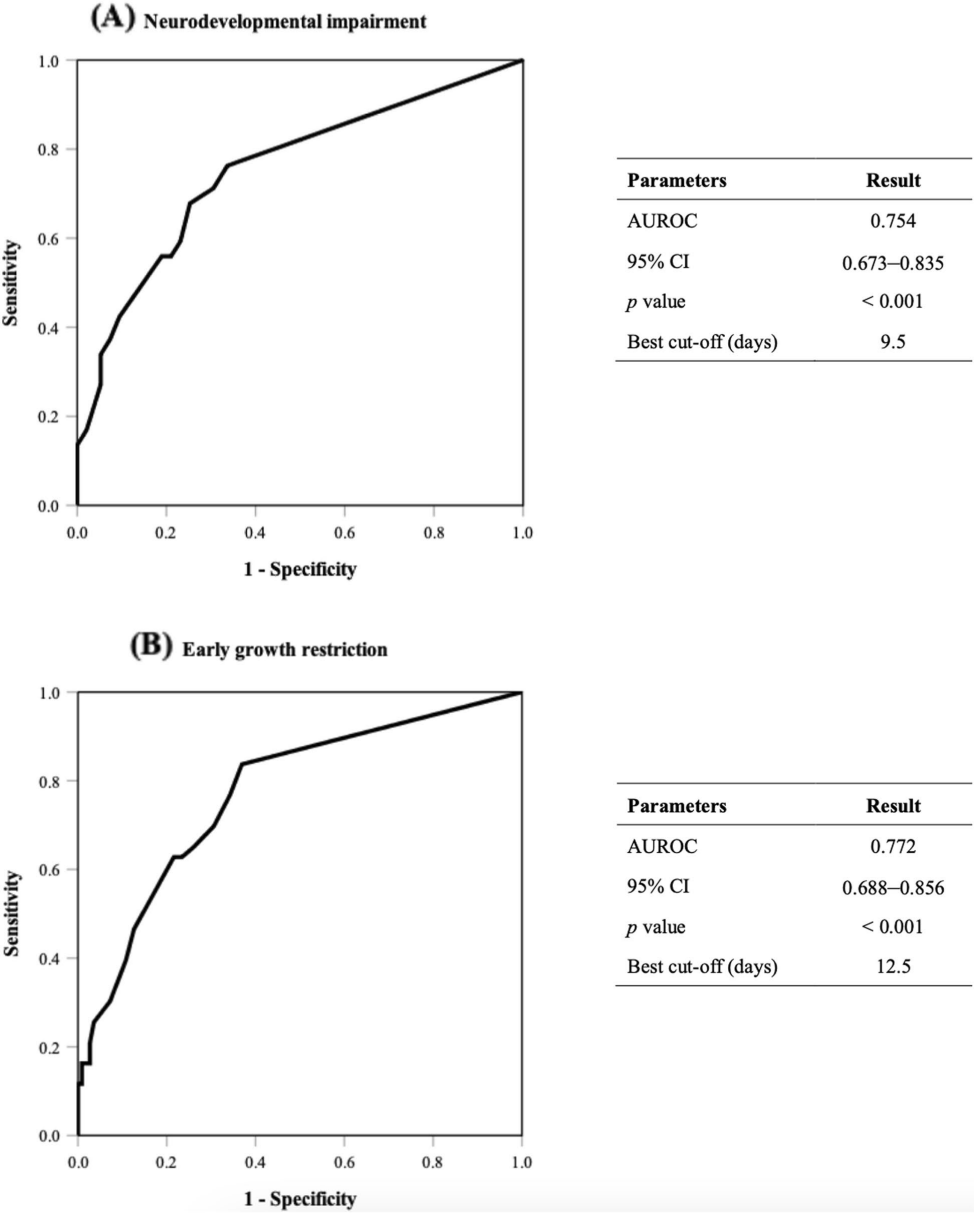
Predictive power of the relationships between the duration of AKI and onset of NDI and EGR in neonates. AKI: Acute kidney injury; NDI: Neurodevelopmental impairment; EGR: Early growth restriction; AUROC: Area under the receiver operating characteristic curve; CI: Confidence interval

To understand why the duration of AKI best predicted NDI and EGR, receiver operating characteristic curves were plotted, as shown in Fig. 2. Figure 2A indicates that the AUROC of the AKI duration predicting NDI was 0.754 (0.673–0.835, *p* < 0.001). Figure 2B shows that the AUROC of AKI duration for predicting EGR was 0.772 (0.688–0.856, *p* < 0.001). The best cut-off duration of AKI for predicting the development of NDI and EGR was 9.5 and 12.5 days, respectively.

## Discussion

This is a study conducting a 13-year analysis of discharged babies diagnosed with severe SAKI. It investigated the association between SAKI and subsequent NDI and EGR in neonates. Our findings confirm that SAKI is a strong independent risk factor for both NDI and EGR. Additionally, we observed that an extended duration of SAKI (exceeding 9.5 days) following birth significantly increases the likelihood of future neurological, growth, and developmental challenges. The area under the receiver operating characteristic curves (AUROCs) for the duration of AKI predicting NDI and EGR were both above 0.7, indicating good predictive performance. These results suggest that SAKI is a critical predictor of neurodevelopmental and growth outcomes in neonates with severe AKI admitted to the NICU.

Previous studies of neonatal AKI often refer to the definitions of Carmody et al. [30] and E. T. Rhone et al. [31]. SCr is typically regarded as the “gold standard” biomarker for clinically diagnosing AKI. However, there are two drawbacks when using SCr as a biomarker in infancy: first, muscle mass, which varies between preterm and term neonates, significantly influences SCr levels [15] and second, during the first 1–2 weeks after birth, the SCr levels of neonates primarily reflect their mother’s SCr [7]. Typically, neonatal SCr levels stabilize within a few days or weeks [7]. Therefore, the diagnosis of neonatal AKI necessitates careful interpretation. In this study, we adhered to the consensus AKI definition of Selewski [8], and modified the neonatal KDIGO AKI definition [2, 3, 6, 13]. We diagnosed neonatal AKI based not only on SCr, but also on changes in UO. This approach is more objective, particularly in comparison to relying solely on SCr levels at a specific time point.

Significantly, BPD is a chronic lung disease in neonates and represents one of the most common and severe consequences of preterm birth. Jensen, Dysart, Gantz, et al. reported that adverse neurodevelopment and growth restriction are common among infants with increased BPD severity [18]. However, no significant difference was found between the SAKI and control groups in our study. This might be due to several reasons: firstly, the small number of severe BPD cases in our study might have lessened the apparent impact of BPD; secondly, the proportion of preterm infants and the gestational age of neonates were very similar between the SAKI and control groups, potentially minimizing the effects of BPD. Our results presumed that SAKI in neonates may be an independent risk factor for NDI and EGR, aside from severe BPD.

While the impacts of perinatal asphyxia, hypoxic ischemic encephalopathy, and IVH on brain damage and potential long-term sequelae are recognized [19–21, 23, 24, 32], our study introduces a new dimension. When accounting for these multiple confounders, SAKI was revealed as a significant independent factor associated with NDI and EGR among neonates in NICUs. This pivotal finding could substantially affect clinical practice. Moreover, optimal nutritional support following discharge from the neonatal care center is crucial for promoting catch-up growth and ensuring favorable long-term development and health [33–36]. In Taiwan, regular visits to the comprehensive national immunization program involve standard measurements of BH, BW, and HC [29]. While growth restriction is a multifaceted, our findings indicate a potential negative influence of SAKI on neonatal growth.

As the survival rate of critically ill newborns improves, it is becoming crucial to consistently monitor their long-term growth and development, with a focus on their neurological development [19–21, 23, 24, 32]. Newborns, especially premature babies who have experienced AKI, may be at higher risk of developing persistent kidney dysfunction, hypertension, and microalbuminuria later in life [6]. Chaturvedi et al. recommended that patients with a history of AKI undergo annual evaluations of blood pressure and urine albumin excretion, even if they appear to be in good health [37]. However, there is no follow-up program specifically for neonates who have previously experienced AKI.

Despite the significant findings of our study, several study limitations should be acknowledged. First, owing to its retrospective and single-center nature, the identification of causal relationships is not feasible. Additionally, while cognition, language, and motor skills were assessed using the Bayley-III scale, and anthropometric characteristics such as BH, BW, and HC were measured, we recognize certain limitations. Relying solely on average values for comparisons between the SAKI and control groups might introduce bias. Furthermore, while these measurements and developmental milestones are informative, they may not sufficiently determine their importance or relevance in a clinical or medical context. Future research involving a large sample size from multiple centers is needed for further clarification of our findings. Beyond the Bayley-III scale and clinical growth indices, the incorporation of additional imaging and laboratory parameters could provide a more comprehensive assessment of growth, development, and kidney health. Lastly, external factors such as the quality of neonatal home care, family resources and support, and economic capacity significantly impact neurodevelopment and nutritional support. These variables should be considered when interpreting our findings.

## Conclusions

The occurrence of SAKI in full- and preterm neonates admitted to NICUs can potentially result in adverse growth and neurological outcomes later in life. Therefore, implementing comprehensive monitoring and optimal interventions should be considered integral components of the management of this pediatric population as they grow and develop.

## Data Availability

The datasets analyzed during the current study are not publicly available because the data are part of the patients’ medical record and are treated as confidential. A completely de-identified version of the data is available from the corresponding author on reasonable request, following approval of the institutional review board.

## Abbreviations

AKI: Acute kidney injury
CA: Corrected age
EGR: Early growth restriction
IVH: Intraventricular hemorrhage
LOS: Length of stay
NICU: Neonatal intensive care unit
NDI: Neurodevelopmental impairment
PMA: Postmenstrual age
RRT: Renal replacement therapy
SAKI: Sustained acute kidney injury
